# Oral Dysbiosis and Systemic Diseases: A Two-Way Relationship?

**DOI:** 10.3390/medicina59111933

**Published:** 2023-11-01

**Authors:** Massimo Pisano

**Affiliations:** Department of Medicine, Surgery and Dentistry “Scuola Medica Salernitana”, University of Salerno, 84084 Salerno, Italy; pisano.studio@virgilio.it

The human body consists of its own cells, but also of microorganisms that are found both inside and outside the human body [1].

Commensal microorganisms are of fundamental importance to the host, as they perform important tasks, such as contributing to host health, counteracting pathogenic bacteria, contributing to the regulation of host homeostasis and balance, and modulating the immune response [2].

The oral microbiota is an important constituent of the human microbiota, with a fundamental role in human health. It is a complex matter, primarily because of the conformation of the oral cavity, which appears to be formed by a set of different ecological niches (tooth, palate, tonsil, saliva, etc.), with very different microbiological habitats that expand and vary greatly in pathology [3].

The main phyla present in the oral cavity and constituting approximately 94% of the oral microbiome are Firmicutes, Bacteroidetes, Proteobacteria, Actinobacteria, Spirochaetes, and Fusobacteria. The remaining 6% of the bacterial phyla consist of Saccharibacteria, Synergistetes, SR1, Gracilibacteria, Chlamydia, Chloroflexi, Tenericutes, and Chlorobi (the detailed list of oral bacteria is available in the Human Oral Microbiome Database, available at http://www.homd.org, accessed on 21 September 2023) [3,4].

Therefore, in the oral cavity, we have an almost borderline microbiota, with representative and characteristic constituent aspects and an apparent scarcity of richness and diversity, although it is in continuous contact with external microbiological agents. External microbial agents, if pathogenic, condition the disease state, and the pharmacological interventions, albeit targeted, can upset the microbiological balance of even the saprophytic flora [5,6].

These microorganisms are often organised in communities, referred to as biofilms. The presence of these biofilms induces an inflammatory host response that leads to the development and progression of periodontal disease [7]. Oral dysbiosis, as in the case of periodontal disease, an inflammatory disease of the periodontium, could be associated with numerous systemic diseases, such as chronic inflammatory or degenerative diseases (e.g., cancer, obesity, diabetes, atherosclerosis, and Alzheimer’s disease) [8,9,10].

Various bacterial species, such as Streptoccoccus mutans, are instead implicated in the development of tooth hard tissue pathologies that, in advanced states, can also compromise pulpal tissue, inducing acute and chronic inflammatory processes [11,12]. It is therefore necessary to treat these teeth endodontically in order to remove the bacteria present within the root canals. Enterococcus faecalis is the species most represented in endodontically treated teeth that do not heal [13]. The shaping and cleansing phases are therefore fundamental in order to break down the intracanal bacterial component [14]. These conditions develop due to an alteration in the oral microbiota which, like the other microbiota present in the human body, contributes to the maintenance of the host’s health.

In fact, under certain conditions, such as in immunocompromised individuals, microorganisms, usually commensals, that are not pathogenic to the host can contribute to the onset of systemic diseases or aggravate already existing pathological conditions [15,16,17].

Several authors have related systemic diseases to conditions of oral dysbiosis and vice versa [18]. Thus, there may be a bidirectional relationship between the oral microbiota and systemic diseases; indeed, the presence of an oral microbiota specifically associated with systemic diseases seems to be, to some extent, a clinical biomarker, although further studies are needed to confirm these associations. However, if this clinical mechanism is confirmed, the early prediction of systemic diseases using microbial detection could be a crucial breakthrough [19]. There are several fields of medicine, such as omics sciences, whose object of study is the characterisation and quantification of pools of biological molecules in order to delineate the structure, functions, and dynamics of an organism, and which are focusing their research on this aspect. In this sense, the oral cavity represents a district of easy application due to its accessibility, which has favoured advances in the use of saliva in diagnostics, the analysis of which could allow the detection of both dental and systemic conditions of dysbiosis [20,21].

Saliva-based biomarkers are useful in the diagnosis of several viral infections such as hepatitis A virus, hepatitis B virus, hepatitis C virus, human immunodeficiency virus (HIV) 1, etc. [22].

Several authors report that in the first 1,000 days after conception, a eubiosis framework is of fundamental importance for the development and maturation of the immune system, with even long-term effects on the health of the unborn child. An altered microbiota is in fact, according to multiple investigations, a predisposing factor for the onset of respiratory, allergic, immune, and metabolic diseases [23].

In addition, several aids have been proposed to restore or prevent dysbiosis, including probiotics [24], the term probiotic being coined from the preposition pro (“in favour of”) and the Greek adjective βιωτικóς (“biotic”), meaning (“life”). The most commonly used probiotics for oral dysbiosis conditions include Bifidobacteria and Lactobacilli [25,26,27].

Ozone, which is widely used in medicine, also has a valid application for modifying the oral microbiota, in particular to eliminate bacteria and fungi, inactivate viruses, and control bleeding. [28].

In conclusion, the aim of this research topic is to provide readers with up-to-date data on the association between dysbiosis of the oral microbiota and systemic conditions [29,30]. We hope that the knowledge gained from these published articles will be useful to initiate new studies in this exciting area where detailed or acquired molecular mechanisms remain unexplored.
